# Photoperiod modulation and hormonal application influence flowering, agronomic, morphological and tuber quality traits in greater yam (*Dioscorea alata* L.)

**DOI:** 10.3389/fpls.2026.1773620

**Published:** 2026-04-13

**Authors:** Komivi Dossa, Erick Malédon, Christophe Perrot, Elie Nudol, Lévy Laurent, Marie-Claire Gravillon, Sandrine Adypain, Olivier Hubert, Hanâ Chaïr

**Affiliations:** 1 CIRAD, UMR AGAP Institut, Petit Bourg, Guadeloupe, France; 2 UMR AGAP Institut, Univ Montpellier, CIRAD, INRAE, Institut Agro, Montpellier, France; 3 CIRAD, Neufchâteau Station, Capesterre-Belle-Eau, Sainte-Marie, Guadeloupe, France; 4 CIRAD, UMR AGAP Institut, Montpellier, France

**Keywords:** *Dioscorea alata*, flowering induction, photoperiod, plant breeding, tuber quality

## Abstract

**Introduction:**

Greater yam (*Dioscorea alata* L.) is a vital staple crop in tropical regions, yet its genetic improvement is severely hampered by poor, irregular, or non-flowering in many genotypes. This flowering recalcitrance creates a major bottleneck for conventional breeding. This study aimed to develop methods for flowering induction in *D. alata* using photoperiod modulation and hormonal applications, and to evaluate the resulting agronomic, morphological and tuber-quality responses.

**Method:**

Over two consecutive seasons, thirteen genotypes with varying flowering abilities were subjected to short-photoperiod (SP), long-photoperiod (LP), and a range of hormonal treatments in a field-based experiment.

**Result:**

We show that photoperiod influences flowering induction in *D. alata*, with strong genotype-dependent responses. The SP treatment was highly effective, successfully inducing fertile, viable flowers in up to 75% of previously non-flowering male and female genotypes. Conversely, the LP treatment completely inhibited flowering but served as a novel tool for synchronizing flowering time among diverse genotypes upon its cessation. A clear trade-off was observed: LP promoted vegetative growth at the expense of tuber yield, whereas SP enhanced both flowering and tuber biomass. While hormonal applications failed to induce flowering, they significantly modulated tuber quality. Notably, the ethylene inhibitor silver thiosulfate increased tuber amylose content, whereas jasmonates significantly reduced it, revealing a link between stress signaling and starch metabolism.

**Conclusion:**

Our findings provide useful insights for yam breeding and suggest practical approaches to induce and synchronize flowering, potentially helping to unlock the vast genetic diversity present in yam germplasm for crop improvement.

## 1 Introduction

Yams (*Dioscorea* spp.) are staple tuber crops that play a vital role in food security, nutrition, and income generation in tropical and subtropical regions (Asiedu and Sartie, 2010). Among cultivated species, *Dioscorea alata* L. (greater yam) stands out for its broad adaptability and high yield potential (Cormier et al., 2021; Wu et al., 2019). However, unlike cereals and fruit crops, yams are predominantly vegetatively propagated (Mondo et al., 2020), and their reproductive biology has long been neglected in breeding efforts. This has significantly hindered genetic gains and the development of improved varieties tailored to specific agroecological conditions and user preferences (Dossa et al., 2026).

In most vegetatively propagated crops, flowering is not directly linked to economic yield, which has led to inadvertent selection against flowering ability during centuries of domestication (Denham et al., 2020). This phenomenon is particularly evident in yams, where a substantial proportion of genotypes in germplasm collections either do not flower or flower irregularly (Mondo et al., 2020). Moreover, the dioecious nature of yams, requiring the co-occurrence of compatible male and female genotypes, adds further complexity to hybrid development Cormier et al. (2021). Even when flowering occurs, it is often sparse and asynchronous (Mondo et al., 2020), impeding controlled crossing and limiting the size and diversity of breeding populations. As a result, a large portion of the genetic diversity along with potentially valuable alleles associated with advantageous traits such as short growth cycle, disease resistance, and high tuber yield and quality remains inaccessible and unexploited in breeding programs (Asiedu and Sartie, 2010). Also, a strong male-biased sex ratio in yam germplasm severely limits controlled crossing. Understanding the physiological and molecular determinants of flowering in yam is therefore essential, not only to elucidate its complex reproductive biology, but also to enable the induction of flowering in non-flowering genotypes, thereby unlocking the full potential of yam breeding programs.

Recent advances in related crops such as cassava have demonstrated the effectiveness of agronomic and physiological interventions in enhancing flowering (Santos et al., 2023; Rodrmguez et al., 2023). Studies have shown that photoperiod extension (Pineda et al., 2020), pruning, and exogenous application of phytohormones such as silver thiosulfate (STS), gibberellic acid (GA), 6-benzylaminopurine (BAP), and ethylene inhibitors can significantly improve flower induction, floral intensity, and sex ratio (Pineda et al., 2020; Hyde and Setter, 2022; Hyde et al., 2024). Similar techniques for overcoming flowering recalcitrance have been successfully explored in other vegetatively propagated root and tuber crops, notably in sweet potato and potato (Almekinders and Wiersema, 1991; Afolabi et al., 2014). In sweet potato, several studies have demonstrated that exogenous application of synthetic auxins such as 2,4-dichlorophenoxyacetic acid (2,4-D) can induce flowering in otherwise non-flowering genotypes (Mutasa et al., 2013). For instance, treatment with 40 ppm 2,4-D significantly increased flower initiation and development, even under suboptimal photoperiod conditions (Watanabe et al., 2016). In potato, which is a long-day plant, the use of extended photoperiod (16–18 hours of light per day) has proven effective in inducing flowering and promoting synchrony among genotypes (Lozoya-Saldana and Miranda-Velázquez, 1987). Kaur et al. (2014) demonstrated that photoperiod extension, when combined with the application of GA and cytokinins, induced flowering in more than 75% of non-flowering genotypes under controlled conditions (Kaur et al., 2014). Similarly, the application of STS, an ethylene inhibitor, under long-day conditions increased floral bud development in several elite cultivars (Almekinders and Struik, 1996). More recently, methods combining pruning, GA_3_ spray, and photoperiod extension were reported to enhance both flowering intensity and reproductive success in diverse potato and cassava lines (Osnato et al., 2022; Kittipadukal et al., 2012; Baguma et al., 2023). These findings support the idea that exogenous hormonal treatments and light regime modulation can bypass physiological flowering constraints and promote reproductive development in clonally propagated crops. These approaches provide a valuable framework for similar efforts in Greater yam.

**Figure 1:**
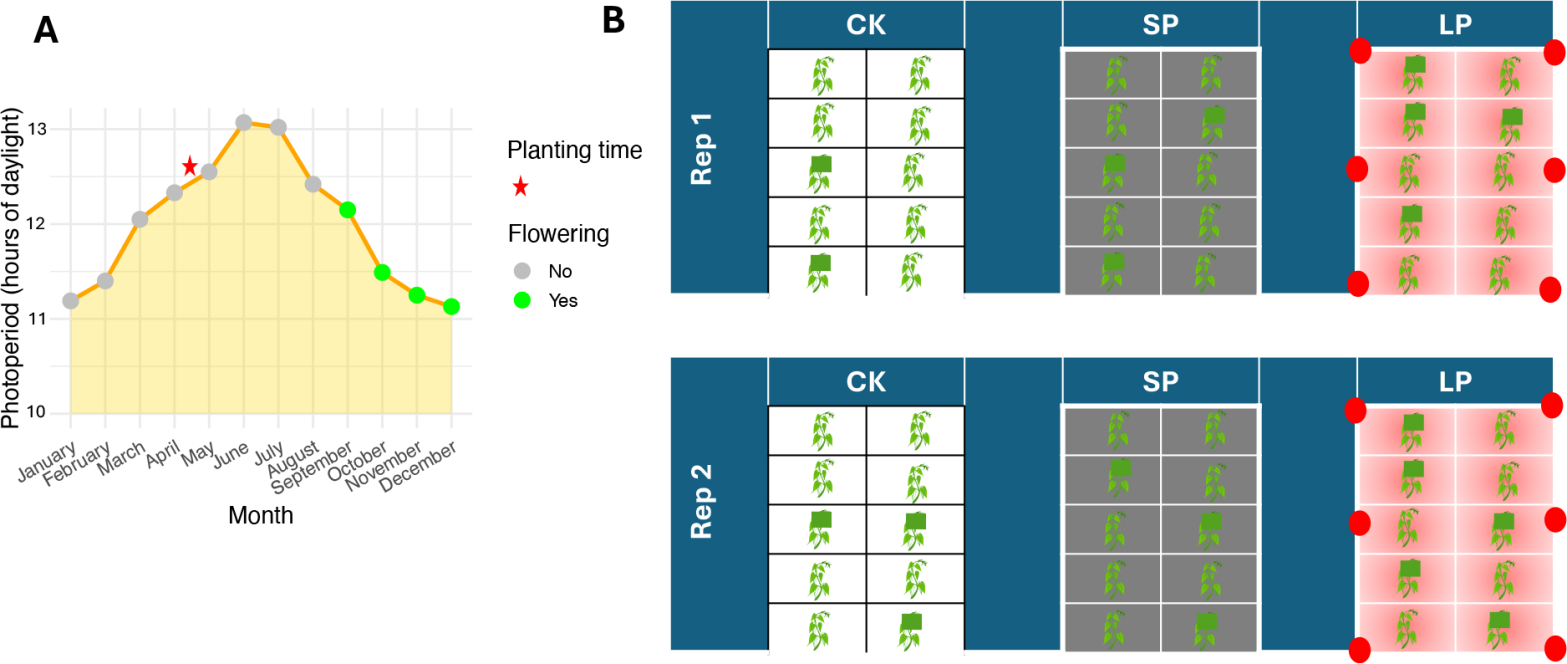
Photoperiodic context and simplified experimental design. **(A)** Relationship between natural photoperiod, planting, and yam flowering time in Guadeloupe. The graph shows the annual variation in day length (orange line). Planting (red star) occurs during increasing day length, while natural flowering (green circles) is initiated as the day length decreases after the summer solstice, confirming a short-day response. **(B)** Schematic view of the experimental layout, illustrating only the photoperiod treatments and five plants per replicate for clarity. The experiment was conducted with two independent replicates. In each replicate, treatments were applied as independent main plots. Photoperiod treatments included the control (CK, natural day length), short photoperiod (SP, achieved by covering plants with an opaque tarp to reduce day length), and long photoperiod (LP, achieved by supplemental artificial lighting to extend day length). The LP treatment was spatially isolated to prevent light contamination. Additional main plots consisted of the different hormonal treatments, which were applied independently of the photoperiod treatments.

In yam so far, mainly *D. rotundata* has received much attention on this topic. Denadi et al. (2024) reported that jasmonic acid and methyl jasmonate applications increased flower intensity and shifted the sex ratio towards femaleness in monoecious plants (Denadi et al., 2024). Mondo et al. (2023) demonstrated that STS not only enhanced floral output but also increased the proportion of female flowers, with additional effects on tuber dry matter content, suggesting pleiotropic or linked physiological responses (Mondo et al., 2023). However, none of these interventions has succeeded in inducing flowering in strictly non-flowering genotypes. In *D. alata*, the effects of photoperiod and exogenous hormones on flowering remain poorly understood (Cormier et al., 2021). The species appears to respond to decreasing day length, as flowering typically occurs several weeks after the summer solstice in most growing regions. In Guadeloupe, for example, planting typically takes place in April–May to coincide with the onset of the rainy season, while flowering peaks in September–October, following a natural decline in day length after July–August (**Figure 1A**). This seasonal pattern suggests that *D. alata* is a short-day plant. Yet, even under identical environmental conditions, considerable intra-varietal variation in flowering behavior has been observed, implying that other factors such as genetic background, soil conditions, and possibly associated microbiomes may modulate floral induction and fertility as reported in other crops (Hill and Li, 2016; Gen-Jiménez et al., 2023; Lee et al., 2024; Maple et al., 2024; Xiong et al., 2021).

Given the critical need to overcome reproductive barriers in yam breeding, the objective of this study was to investigate the effects of photoperiod modulation and exogenous hormonal applications on flowering behavior in *D. alata*. Specifically, we aimed to evaluate the influence of these treatments on flower initiation, flowering intensity, sex expression, and synchrony. In addition, we assessed the impact of these interventions on agronomic and morphological traits and tuber quality parameters to determine potential trade-offs or synergies. This work contributes to the growing body of knowledge on yam reproductive biology and provides practical tools for accelerating hybridization and selection in breeding programs.

## 2 Materials and methods

### 2.1 Plant materials and experimental site

The study was conducted over two consecutive agronomic seasons (2022–2023 and2023–2024) at the CIRAD Roujol experimental station in Guadeloupe (16°10′ 56″ N, 61° 35’ 24” W; 10 m above sea level). The soil at the site is of the alluvial type, with detailed characteristics provided in **Supplementary Table 1**. Weather parameters during the experimental periods are presented in **Supplementary Figure S1**.

Thirteen greater yam genotypes (*Dioscorea alata* L.) from the Biological Resource Center of Tropical Plant (INRAE) and CIRAD yam collections (Cormier et al., 2021) were used in this study (**Table 1**). These included three genotypes with a history of regular flowering (CIRAD121, CIRAD133, CIRAD120), which served as positive controls, and ten genotypes with irregular or no flowering history, which were the subjects of the induction treatments.

**Table 1:** List and characteristics of the *Dioscorea alata* genotypes used in the flowering induction experiments.

Accession code	Flowering	Sex phenotype	Local name	Cycle length
CIRAD121	Regular	F	74F	long
CIRAD133	Regular	F	Boutou	long
CIRAD153	Irregular	M	Kabusa	long
CIRAD170	No		Sinoua	short
CIRAD139	No		Florido	long
CIRAD140	Irregular		Goana	long
CIRAD130	Irregular		Belep	long
CIRAD138	No		Fénakué	long
CIRAD120	Regular	M	14M	long
CIRAD163	No		Pakutrany	long
CIRAD173	No		Toki	short
CIRAD154	No		Kinabayo	long
CIRAD175	No		Wassa	short

The thirteen genotypes were selected from the CIRAD collection based on their historical flowering behavior. The set includes ten genotypes with irregular or no flowering status, which served as the primary test subjects, and three genotypes with regular flowering, which were used as positive controls.

The experiment was conducted as a series of independent trials (**Figure 1B**, **Table 2**). Two photoperiod treatments (short photoperiod, SP; long photoperiod, LP) and hormonal treatments were applied separately. For each treatment, 10 yam genotypes were evaluated, with 30 plants per genotype arranged in 2 replicates of 15 plants. Plots receiving different treatments were spatially separated (3 m, and 5 m for the long photoperiod treatment) to prevent interference, particularly light contamination.

**Table 2:** Summary of the genotypes tested, treatments applied, and experimental seasons.

Genotypes	Treatments	Seasons
CIRAD153	Long photoperiod (LP)	2022-2023
CIRAD170		
CIRAD139	Short photoperiod (SP)	
CIRAD175		
CIRAD130	Control (CK)	
CIRAD138		
CIRAD163	Gibberellic acid (GA; 200 mg/L)	
CIRAD173		
CIRAD133	6-benzylaminopurine (BAP; 400 mg/L)	
CIRAD120		
	Silver thiosulfate (STS; 1 mM)	
	Spermidine (Spd; 1.45 mg/L)	
CIRAD153	LP	2023-2024
CIRAD139		
CIRAD140	SP	
CIRAD170		
CIRAD138	CK	
CIRAD163		
CIRAD130	GA; 200 mg/L	
CIRAD154		
CIRAD121	STS; 50 mM	
CIRAD120		
	Ethephon (Ethrel; 400 µL/L)	
	Methyl jasmonate (MEJA; 1 mM)	
	Jasmonic acid (JA; 1 mM)	
	Indole-3-acetic acid (IAA; 1 mM)	

The field was prepared by creating ridges (50 m long and 2 m apart), which were covered with paper mulch to suppress weed growth. Plants were spaced 30 cm apart within ridges, with genotypes separated by 1 m. The plot was managed under drip irrigation.

Seed tubers (approx. 100 g) were planted on May 10th, 2022, for the first season and May 21st, 2023, for the second. Prior to planting, tubers were disinfected by immersion for 30 seconds in a solution containing 10 L of water, 0.5 L of black soap, and 0.5 L of alcohol. Tubers were harvested at full plant senescence, which occurred in February of the following year for each season. After harvest, tubers were cured and stored at 27 °C.

### 2.2 Flowering induction treatments

Treatments were applied during the natural flowering window for *D. alata* in Guadeloupe and were selected based on previous literature in yam and other crops (**Table 3**).

**Table 3:** Rationale for the selection of photoperiod and hormonal treatments for flowering induction.

Treatments	Reported effect	References
Gibberellic acid (GA)	Promotes floral initiation and stem elongation. Widely used to induce flowering in long-day plants and can overcome flowering barriers in various species, including cassava.	(Tang et al., 1983)
6-benzylaminopurine (BAP)	cytokinin that promotes cell division and shoot proliferation. In some species like cassava, it can induce flowering and influence sex expression, often promoting femaleness.	(Tang et al., 1983); (Pineda et al., 2020)
Silver thiosulfate (STS)	An ethylene inhibitor. By blocking the action of ethylene, it can prevent flower abscission and promote flowering in species where ethylene is an inhibitory signal. It has been shown to enhance floral intensity in cassava and white yam.	(Hyde et al., 2020) (Mondo et al., 2023)
Spermidine (Spd)	A polyamine involved in various plant developmental processes. Exogenous application has been shown to promote floral induction and improve fruit set in horticultural crops like apple.	(Qin et al., 2019)
Ethrel (Ethephon)	An ethylene-releasing compound. Ethylene is a key regulator of sex expression in cucurbits, where it strongly promotes the development of female flowers. It can also induce flowering in some families (e.g. Bromeliaceae).	(Sadhu and Das, 1978); (Li et al., 2021)
Methyl Jasmonate (MEJA)/Jasmonic acid (JA)	Plant stress and defense hormones. In white yam (*D. rotundata*), application of jasmonates was reported to increase the intensity of flowering and shift the sex ratio towards femaleness in monoecious plants.	(Denadi et al., 2024)
Short photoperiod (SP)	An environmental cue that induces flowering in short-day plants (SDPs) like soybean, rice, and many tropical species. The decreasing day length signals the transition from vegetative to reproductive growth	(Thomas and Vince-Prue, 1996)
Long photoperiod (LP)	An environmental cue that induces flowering in long-day plants like potato and Arabidopsis. Conversely, it inhibits flowering in SDPs, maintaining them in a vegetative state.	(Wang et al., 2021); (Yeang, 2013)

In the 2022–2023 season, ten genotypes (CIRAD153, CIRAD170, CIRAD139, CIRAD175, CIRAD130, CIRAD138, CIRAD163, CIRAD173, and the two checks CIRAD133 and CIRAD120) were evaluated. From September 1^st^ to October 28^th^, 2022, plants were subjected to seven different treatments. These included foliar applications of gibberellic acid (GA; 200 mg/L), 6-benzylaminopurine (BAP; 400 mg/L), silver thiosulfate (STS; 1 mM), and spermidine (Spd; 1.45 mg/L). This experiment also included a short photoperiod (SP) treatment, LP treatment, and a control (CK) where plants were sprayed with demineralized water containing a surfactant (**Table 2**).

For the 2023–2024 season, the experimental design was adjusted based on the first year’s observations. Ten genotypes were tested, including a revised set of test genotypes (CIRAD153, CIRAD139, CIRAD140, CIRAD170, CIRAD138, CIRAD163, CIRAD130, CIRAD154) and two positive checks (CIRAD121, and CIRAD120). During the same application window from September 1^st^ to October 28^th^, 2023, the hormonal applications were updated to include gibberellic acid (GA; 200 mg/L), a higher concentration of silver thiosulfate (STS; 50 mM), ethephon (Ethrel; 400 µL/L), methyl jasmonate (MEJA; 1 mM), jasmonic acid (JA; 1 mM), and indole-3-acetic acid (IAA; 1 mM). The SP, LP, and CK treatments were maintained as in the previous season (**Table 2**). In the 2022–2023 season, STS was applied at 1 mM as an exploratory concentration. Because limited physiological responses were observed, the concentration was increased to a high concentration (50 mM) in the following season (2023–2024) in order to test whether a stronger inhibition of ethylene perception could influence flowering or tuber traits.

### 2.3 Hormonal application and photoperiod modulation

#### 2.3.1 Phytohormone application

All hormonal solutions were freshly prepared on the day of application, and Tween-20 (0.1% v/v) was added as a surfactant to ensure adhesion and coverage. Treatments were applied as a foliar spray every Monday, Wednesday, and Friday between 8:00 and 10:00 a.m. Approximately 10 mL of solution was sprayed per plant, ensuring the entire plant, with an emphasis on leaves and vines, was thoroughly covered.

#### 2.3.2 Photoperiod modulation

LP: Daylength was extended using six LED RGB projectors (COLIBRI IP66) per replicate, each with a power rating of 50 W and a light intensity of 6000 lumens. The projectors emitted red light with a peak wavelength of 630–640 nm (**Supplementary Figure S2**). Lights were automatically switched on at 5:00 p.m. and off at 6:00 a.m. daily. In the 2023–2024 season, the LP treatment period was extended (August 2^nd^ to November 6^th^, 2023) to further study its effect on flowering delay.

SP: A short-day condition was created by covering plants with an opaque black tarp daily from 3:00 p.m. to 7:00 a.m (**Supplementary Figure S3**). In the 2023–2024 season, the SP treatment was initiated earlier in the plant cycle (August 2^nd^ to September 4^th^, 2023) to test if application prior to the natural flowering window was more effective.

### 2.4 Data collection and measurements

#### 2.4.1 Flowering and fertility assessment

Plants were monitored daily to record the date of first flowering. Flowering intensity and synchrony (observational traits) within and between genotypes were recorded. Flowering intensity was assessed using a semi-quantitative ordinal scale (1 = low; 2 = high) based on the visual evaluation of the abundance of inflorescences per plant within each experimental unit. Observations were conducted across treatments by the same observer to ensure consistency in scoring. The genotype CIRAD121, known for its high flowering intensity, was used as a reference control to facilitate comparisons among treatments and genotypes.

Flowering synchrony was evaluated in the LP treatment by recording the date of first flowering for the two reference genotypes at the end of the treatment. Synchrony was determined by comparing the distribution of flowering onset dates among genotypes. In the control treatment (CK), the two reference genotypes displayed distinct flowering onset dates. Therefore, in the LP treatment, if flowering onset occurred within a narrow time window, the genotypes were considered to exhibit flowering synchrony. For genotypes that successfully flowered under SP treatment, fertility was assessed. Male fertility was evaluated through pollen viability tests using Alexander’s staining solution on 10 flowers. Male plants were considered fertile when the majority of pollen grains (>70%) per slide stained purple/red, indicating viable pollen. Female fertility was evaluated through controlled hand pollinations. For each genotype, 50 pollination attempts were conducted. Successful fertilization was assessed by observing fruit set and subsequent seed formation following pollination.

##### 2.4.1.1 Agronomic, morphological and tuber yield traits

Leaf traits, including chlorophyll content, leaf area and leaf dry matter content, were measured (quantitative traits) during the vegetative growth phase on twelve randomly selected mature leaves, with six leaves from each replicate. Chlorophyll content was estimated non-destructively using a SPAD meter, following the protocol described in Dossa et al. (2025b). Leaf area was determined from scanned images using a custom R script, as detailed at https://github.com/samdossa/yam_leaf_trait_analysis. Leaf dry matter content was determined by oven-drying fresh leaves at 55 °C for 72 h until constant weight was reached. At harvest, tuber yield components were measured for each plant (n=30), including the number of tubers and the total tuber weight per genotype.

#### 2.4.2 Tuber quality analysis

Tuber samples were collected at harvest from the ten evaluated genotypes in each treatment. For each genotype, five tubers were randomly selected to assess quality parameters. Tuber dry matter content was determined by oven-drying a subsample of fresh tuber at 70 °C for 72 h until constant weight was reached. Soluble solids content and amylose content were also measured. For each tuber, measurements were performed with 10 technical replicates.

#### 2.4.3 Soluble solids content (°Brix)

To 100 mg of freeze-dried yam powder in an Eppendorf tube, 500 µL of distilled water was added. The mixture was homogenized by vortexing and then centrifuged at 9,500 RPM for 10 minutes at 20 °C (SIGMA 3-81KS centrifuge). The soluble solids content (°Brix) of the supernatant was measured using a HANNA HI96811 digital refractometer. The final value was corrected by multiplying the measured °Brix by the dilution factor.

#### 2.4.4 Amylose content determination

Amylose content was determined using a spectrophotometric method. For each sample, 100 mg of freeze-dried yam powder was weighed into a 15 mL Falcon tube. While vortexing, 1 mL of ethanol was added, followed by 9 mL of 1 M NaOH. The tubes were sealed and placed on a rotary shaker at 20 rpm for overnight extraction. The following day, the entire content of each tube was quantitatively transferred to a 100 mL volumetric flask and brought to volume with distilled water. The final extracts were stored at 4 °C until analysis. In a 15 mL Falcon tube, 500 µL of the sample extract was added to 5 mL of distilled water. Then, 100 µL of glacial acetic acid was added, and the solution was homogenized by vortexing. Finally, 200 µL of iodine stock solution (containing 2% KI and 0.2% I_2_) and 4.2 mL of distilled water were added, and the mixture was homogenized again. Ten minutes after the final addition of water, 200 µL of each solution was transferred to a 96-well microplate. Absorbance was measured at 545 nm and 620 nm using a Tecan Infinite 200Pro plate reader (Tecan, Männedorf, Switzerland) controlled by i-control software (v1.10.4.0).

Amylose content was calculated based on standard curves prepared for pure amylose and amylopectin (0–1000 µg/mL in 0.09 M NaOH). The concentrations of amylose ([AS]) and amylopectin ([AP]) in the extract were determined by solving the system of two linear equations derived from the Beer-Lambert law, using blank-corrected absorbance values (A) and the slopes of the standard curves (α):


$$A_{620}\;=\;\left(\alpha_{620}\;nm\;AS\;\times \;\left[AS\right]\right)\;+\;\left(\alpha_{620}\;nm\;AP\;\times \;\left[AP\right]\right)$$

$$A_{545}\;=\;\left(\alpha_{545}\;nm\;AS\;\times \;\left[AS\right]\right)\;+\;\left(\alpha_{545}\;nm\;AP\;\times \;\left[AP\right]\right)$$

The final amylose content was expressed as a percentage of the dry flour weight (g/100g).

### 2.5 Statistical analysis

Because the experimental design differed between the two seasons (genotypes evaluated, hormonal treatments, hormone concentrations, and timing of SP application), statistical analyses were conducted independently for each season. In addition, flowering induction treatments (photoperiod modulation and phytohormone applications) were applied independently and not in combination, therefore, statistical analyses were conducted separately for each treatment. For each treatment, the effects on flowering induction, yield, and quality traits were assessed using linear mixed models, with treatment considered as a fixed factor. Genotype and replicate were included as random factors to account for genetic variability and field replication. When significant effects were detected, *post hoc* pairwise comparisons were performed using Tukey’s HSD test. Statistical significance was assessed at p< 0.05. All analyses were conducted in R version 4.4.2 (R Core Team).

## 3 Result

### 3.1 Impact of photoperiod modulation on *Dioscorea alata* traits

Across both experimental seasons, the two positive control genotypes consistently flowered under the control (CK) treatment, whereas none of the eight test genotypes, initiated flowering in the absence of specific interventions.

#### 3.1.1 Flowering induction in 2022–2023

**Figure 2:**
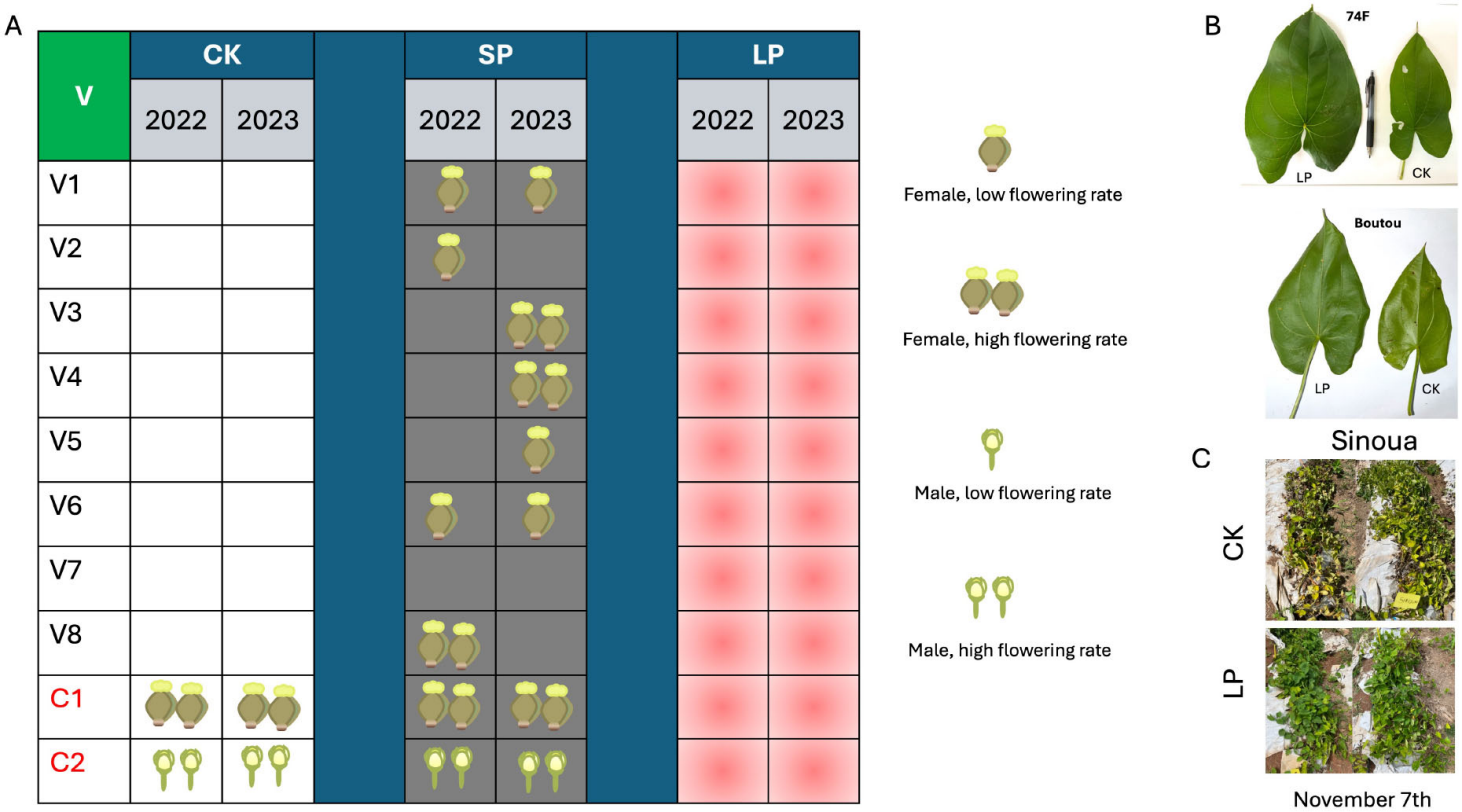
Summary of flowering induction, inhibition, and pleiotropic effects of photoperiod treatments on *Dioscorea alata* genotypes. **(A)** Flowering response of eight test genotypes (V1–V8) and two positive controls (C1–C2) over two seasons (2022 and 2023). White cells indicate no flowering; gray cells indicate successful flowering induction under short photoperiod (SP); red cells indicate flowering inhibition under long photoperiod (LP). Icons represent the sex and intensity of flowering observed. **(B)** Pleiotropic effects of LP on vegetative morphology. Note the significantly larger leaf size in genotypes ‘74F’ and ‘Boutou’ under LP compared to the control (CK). **(C)** Delayed senescence under LP treatment. The genotype ‘Sinoua’ remains vegetatively active and green under LP on November 7th, while the same genotype under CK has already entered senescence.

In the 2022–2023 experiment, flower induction was successfully achieved in 50% of the eight tested genotypes (number of plant n = 30) under the short photoperiod (SP) treatment (**Figure 2A**). Specifically, CIRAD153 (male), CIRAD139 (male), CIRAD130 (male), and CIRAD138 (female) produced flowers. In contrast, the three short-cycle genotypes CIRAD170, CIRAD173, and CIRAD175 did not flower under any treatment (**Figure 2A**). These genotypes entered senescence in October–November, suggesting that treatment application was initiated too late in the cycle to induce flowering.

Interestingly, the positive controls CIRAD120 and CIRAD133 flowered earlier under SP, with flowering occurring 7 and 16 days earlier, respectively, compared to CK.

In contrast, the long photoperiod (LP) treatment inhibited or delayed flowering across all genotypes (**Figure 2A**). Neither of the positive control genotypes flowered under LP. However, they initiated flowering synchronously one week after the end of the LP treatment, reinforcing the hypothesis that *D. alata* is a short-day species. Notably, CIRAD120, which typically flowers in early October, and CIRAD133, which flowers in late October, both flowered within a similar timeframe following LP cessation. These observations suggest that LP may offer a tool to synchronize flowering. The eight test genotypes did not flower after the end of LP.

**Figure 3:**
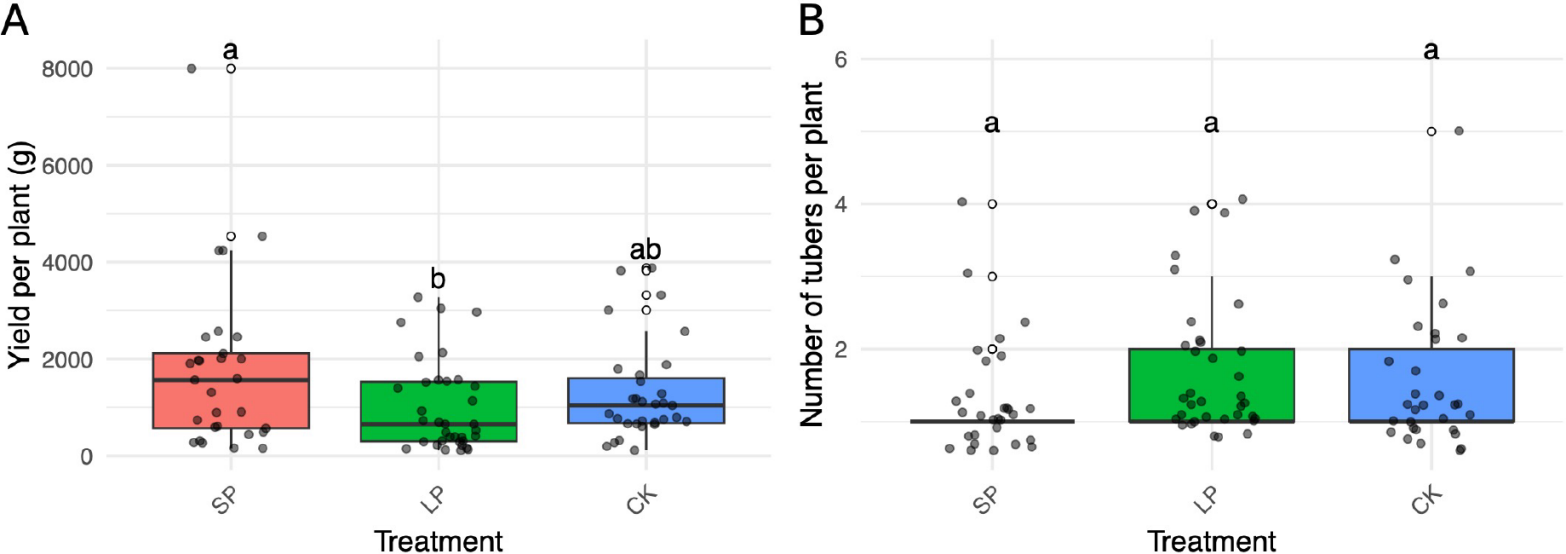
Effects of photoperiod treatments on *Dioscorea alata* yield per plant and tuber number per plant during the 2022–2023 season. Boxplots comparing the effects of short photoperiod (SP), long photoperiod (LP), and control (CK) treatments. **(A)** Tuber yield per plant. The SP treatment resulted in a significantly higher tuber yield per plant than the LP treatment. **(B)** Number of tubers per plant. No significant differences were observed among the treatments. Letters above boxes indicate significant differences between treatments (ANOVA, Tukey’s HSD, p< 0.05). In each treatment, 10 genotypes were tested with 30 plants per genotypes.

Vegetative performance was also markedly affected by LP. Plants grown under LP exhibited increased aerial biomass, with vigorous growth, larger leaves (**Figure 2B**), and delayed senescence lasting until late November (**Figure 2C**). However, this vigorous vegetative growth came at a cost: tuber yield was significantly reduced under LP, whereas SP-treated plants yielded the highest tuber biomass (**Figure 3A**). The number of tubers per plant, however, was not significantly affected by photoperiod treatments (**Figure 3B**).

#### 3.1.2 Flowering induction in 2023–2024

In the following season (2023–2024), the flowering induction rate was 75% under SP for the eight tested genotypes (number of plant n = 30). Flowering was observed in six genotypes: CIRAD153 (male), CIRAD139 (male), CIRAD130 (male), CIRAD140 (female), CIRAD138 (female), and CIRAD154 (female). Two factors may explain this observation: (1) the use of predominantly long-cycle genotypes, and (2) the earlier application of SP treatments, prior to the onset of the natural flowering window.

**Figure 4:**
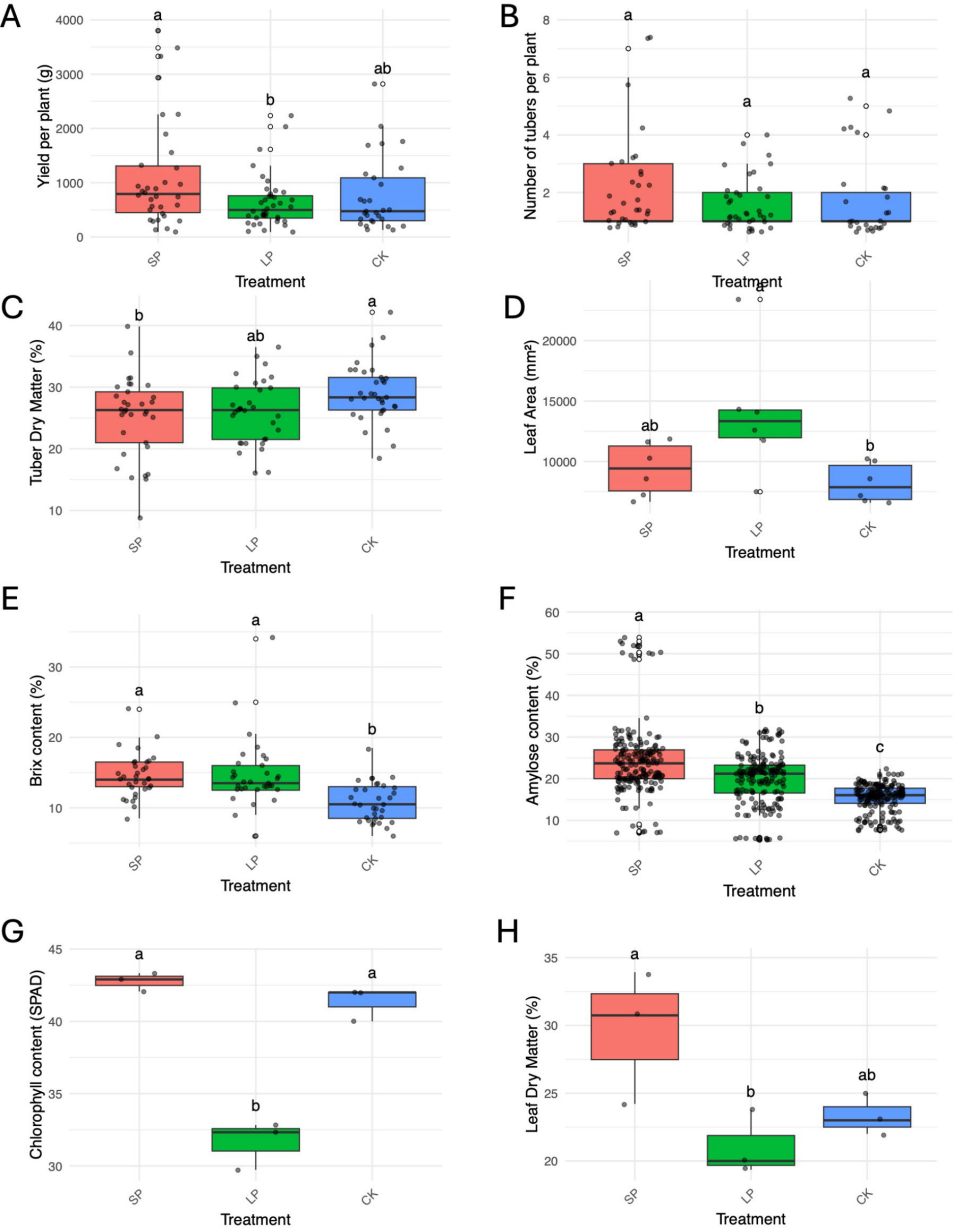
Effects of photoperiod treatments on *Dioscorea alata* agronomic, morphological, physiological, and tuber quality traits during the 2023–2024 season. Boxplots comparing the effects of short photoperiod (SP), long photoperiod (LP), and control (CK) treatments. **(A)** Tuber yield per plant was significantly higher under SP than LP. **(B)** The number of tubers per plant was not affected by the treatments (Ten genotypes and 30 plants per genotypes were evaluated). **(C)** Tuber dry matter was significantly lower in SP-treated plants compared to the control (Ten genotypes were evaluated using five randomly selected tubers per genotype, with ten technical replicates for each measurement). **(D)** Leaf area was significantly increased under LP compared to the control (Ten genotypes and 12 leaves samples per genotype were evaluated). **(E)** Soluble solids content (°Brix) was significantly higher in both SP and LP treatments compared to the control. **(F)** Tuber amylose content showed a significant gradient, with SP > LP > CK (Ten genotypes were evaluated using five randomly selected tubers per genotype, with ten technical replicates for each measurement). **(G)** Leaf chlorophyll content (SPAD) was significantly reduced under LP. **(H)** Leaf dry matter content was significantly higher under SP than under LP. Letters above boxes indicate significant differences between treatments (p< 0.05).

No flowering was recorded under LP. Nonetheless, synchronized flowering occurred in the positive controls, with CIRAD120 and CIRAD121 initiating flowering 11 and 13 days, respectively, after LP termination. Data from the 2023–2024 season revealed that photoperiod modulation fundamentally altered plant resource allocation, creating a distinct trade-off between vegetative and storage organ development (**Figure 4**).

Plants grown under the LP treatment were characterized by enhanced vegetative vigor. They exhibited a significantly larger mean leaf area (mean ≈ 14,000 mm²) compared to the control plants (mean ≈ 9,000 mm²). However, significant reductions in both chlorophyll content (SPAD) and leaf dry matter content were observed when compared to plants in the SP treatment (**Figure 4**).

This prioritization of vegetative growth under LP came at a direct cost to tuber production. The LP treatment resulted in the lowest mean tuber yield per plant (mean ≈ 900 g), which was significantly lower than the yield from SP-treated plants (mean ≈ 1,400 g). SP not only induced flowering but also promoted the highest tuber yield. Despite these large differences in biomass, the total number of tubers produced per plant was statistically similar across all photoperiod treatments.

Tuber quality parameters were also significantly and differentially affected by photoperiod modulation (**Figure 4**). The most striking effect was observed in tuber amylose content, where a clear and statistically significant gradient emerged: SP-treated tubers contained the highest amylose levels (mean ≈ 25%), followed by LP-treated tubers (mean ≈ 18%), while CK tubers had the significantly lowest levels (mean ≈ 13%). In contrast, tuber dry matter was highest in CK plants (mean ≈ 32%) and was significantly reduced under the SP condition (mean ≈ 28%). Soluble solids content (°Brix) was also significantly higher in tubers from both the SP and LP treatments compared to those from the CK group.

Crucially, the flowers induced by the SP treatment were confirmed to be reproductively viable. Pollen viability was confirmed through staining, which showed >70% of pollen grains well-formed, colored pollen grains from induced male flowers like ‘Florido’ (**Supplementary Figure S4A**). Furthermore, successful hand-pollinations were conducted between newly flowering genotypes. These crosses, such as ‘74F’ × ‘Florido’ and ‘Boutou’ × ‘BELEP’, resulted in the development of healthy fruits (40% and 72% successful crosses rate, respectively), confirming female fertility and the potential for generating novel hybrid progeny (**Supplementary Figures S4B**, **C**).

### 3.2 Impact of hormonal applications on *D. alata* traits

While hormonal applications failed to induce flowering in non-flowering genotypes, they had significant and often specific effects on plant morphology, tuber yield, and particularly on tuber quality parameters.

Several hormonal treatments induced distinct, non-flowering morphological changes. Application of 6-benzylaminopurine (BAP) had a strong phytotoxic and inhibitory effect, causing severe leaf crinkling, marginal necrosis, and ultimately blocking flowering even in the positive control genotypes (**Supplementary Figure S4**). In contrast, Gibberellic acid (GA) treatment did not inhibit flowering but altered floral architecture, causing significant elongation and curvature of pedicels compared to the untreated controls (**Supplementary Figure S5**). Jasmonate applications (JA/MEJA) were noted to increase flowering intensity in controls, while Ethrel promoted aerial bulbil formation.

**Figure 5:**
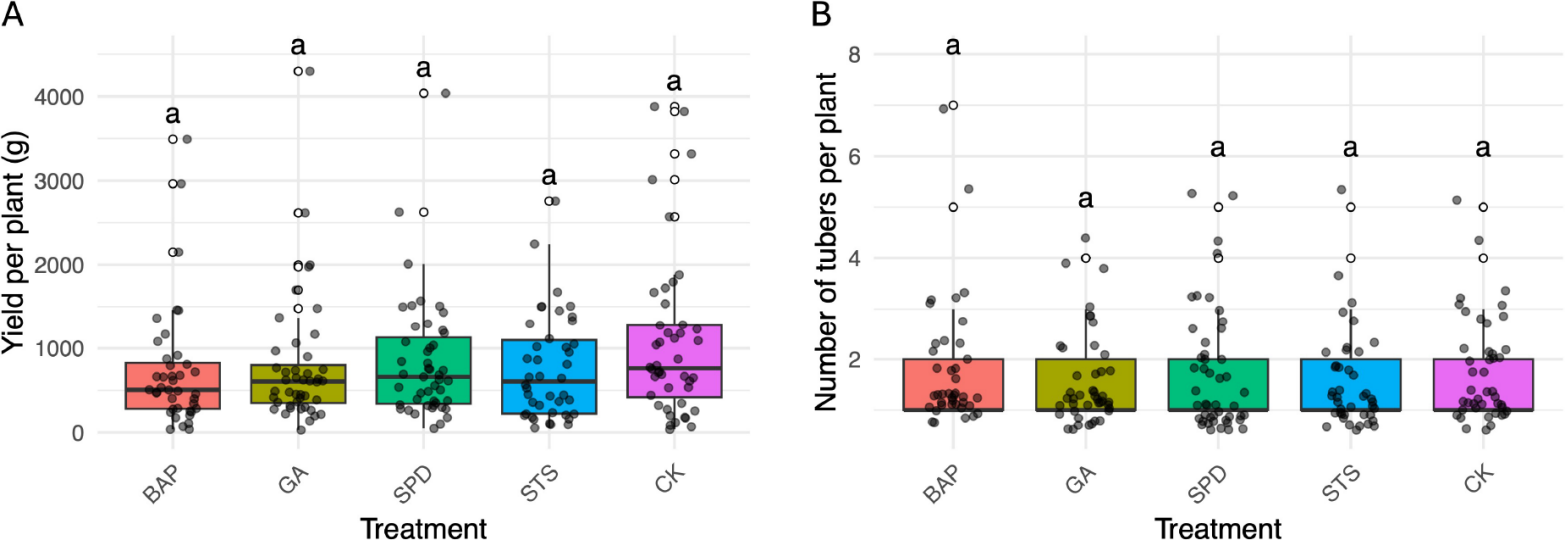
Effects of hormonal treatments on *Dioscorea alata* tuber yield and tuber number per plant during the 2022–2023 season. Neither **(A)** tuber yield per plant nor **(B)** number of tubers per plant were significantly affected by the application of 6-Benzylaminopurine (BAP), Gibberellic acid (GA), Spermidine (SPD), or Silver Thiosulfate (STS) compared to the control (CK). Letters above boxes indicate no significant differences between treatments (p > 0.05). Ten genotypes and 30 plants per genotypes were evaluated.

**Figure 6:**
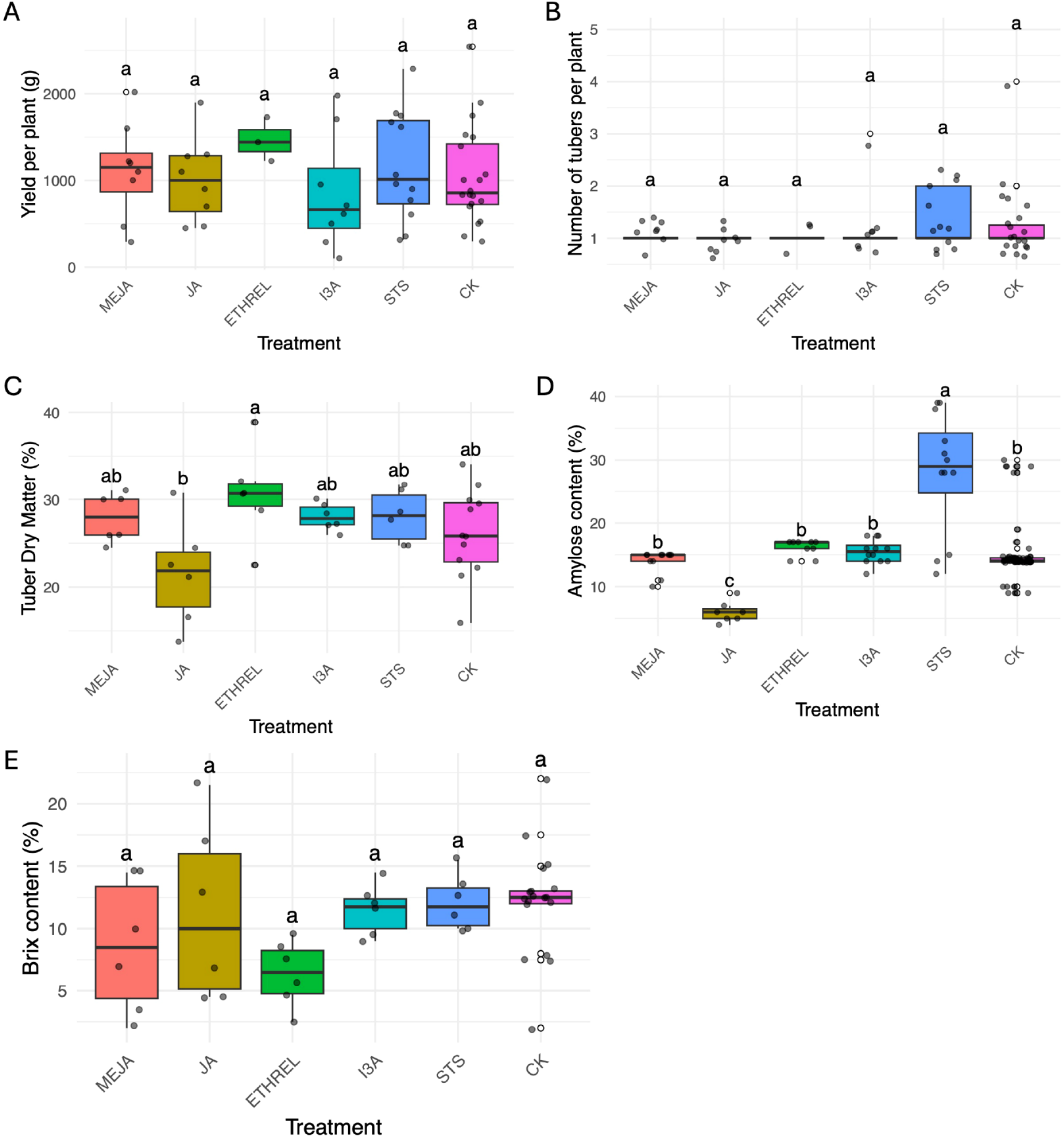
Effects of hormonal treatments on *Dioscorea alata* tuber number and quality parameters during the 2023–2024 season. **(A)** Yield per plant and **(B)** Number of tubers per plant were not significantly affected by hormonal treatments (Ten genotypes and 30 plants per genotypes were evaluated). **(C)** Tuber dry matter content. Jasmonic acid (JA) treatment significantly reduced tuber dry matter compared to Ethrel. **(D)** Tuber amylose content. Silver Thiosulfate (STS) treatment dramatically and significantly increased amylose content, while JA and Methyl Jasmonate (MEJA) significantly reduced it compared to the control. **(E)** Soluble solids content (°Brix). JA treatment resulted in a significantly lower Brix content compared to other treatments (Ten genotypes were evaluated using five randomly selected tubers per genotype, with ten technical replicates for each measurement). Letters above boxes indicate significant differences (p< 0.05).

In the 2022–2023 season, foliar application of BAP, GA, Spermidine, or Silver Thiosulfate (STS) had no significant effect on either tuber yield per plant or the number of tubers per plant when compared to the control group (**Figure 5**). Similarly, in the 2023–2024 season, none of the tested phytohormones (MEJA, JA, Ethrel, I3A, STS) significantly influenced final tuber yield or tuber number relative to the control (**Figures 6A, B**).

In contrast, several hormones profoundly altered tuber quality parameters in the 2023–2024 season (**Figures 6C–E**). STS application caused a highly significant increase in tuber amylose content, reaching a mean of approximately 32%, which was substantially higher than all other treatments. Conversely, both jasmonate treatments significantly reduced starch quality; JA, in particular, resulted in the lowest amylose content (mean ≈ 8%), a nearly four-fold reduction compared to the STS treatment. Furthermore, JA had additional negative impacts on quality, leading to the significantly lowest tuber dry matter content and the lowest soluble solids content (°Brix) among all treatments. Ethrel application was notable for producing tubers with a significantly higher dry matter content than those from the JA treatment.

## 4 Discussion

This study provides evidence that flowering in greater yam, a critical yet challenging trait for genetic improvement, is highly influenced by photoperiod. We show that a short-photoperiod (SP) treatment can reliably induce fertile flowers in previously irregular or non-flowering genotypes, while a long-photoperiod (LP) treatment inhibits flowering but serves as a novel tool for synchronization. Furthermore, while exogenous hormonal applications failed to initiate flowering, they significantly modulated tuber quality, revealing complex physiological crosstalk between reproductive signaling and storage organ metabolism. Because the experimental design evolved between seasons, including differences in genotypes and treatment protocols, cross-season comparisons should be interpreted cautiously and are presented as descriptive trends rather than formal statistical tests.

### 4.1 Photoperiod is the primary gateway to flowering in *D. alata*

The main finding of this work is the confirmation of *D. alata* as a strong short-day response species (SDP), where the photoperiodic pathway appears to be a master regulator of the floral transition. The success of the SP treatment (75% induction rate) in genotypes that hardly flower for years in our germplasm collections is a key finding. This aligns with observations in other yam species like *D. rotundata* and historical suggestions that yam flowering coincides with decreasing day length after the summer solstice (Shiwachi et al., 2005; Ile et al., 2007). The mechanism likely involves the canonical SDP pathway, where short days permit the accumulation of the florigen signal, Florigen T (FT) protein, in the leaves, which is then transported to the shoot apical meristem to initiate flowering (Tsuji et al., 2011, 2013). Our earlier application of SP in the second year was more successful, suggesting a developmental “competence” window where the plant must be receptive to the photoperiodic signal before the onset of natural senescence cues (Serrano-Bueno et al., 2021).

Conversely, the complete inhibition of flowering by the LP treatment provides a proof of this photoperiodic control. In SDPs, prolonged exposure to light, especially red light as used in our experiment, may maintain phytochrome in its active Pfr form. This active state is known to suppress the expression of florigen promoters, effectively blocking the floral transition and maintaining the plant in a vegetative state (Osnato et al., 2022; Takagi et al., 2023). The enhanced vegetative growth, including larger leaves and delayed senescence under LP, is a classic manifestation of this resource allocation away from reproduction (Antonietta et al., 2024). This phenomenon, while detrimental to tuber yield, may offer a practical application. The synchronized flowering observed in control varieties upon cessation of the LP treatment is a key finding for yam. By holding diverse genotypes in a vegetative “holding pattern,” LP treatment can erase natural differences in flowering time, allowing for synchronized floral initiation upon return to a neutral or short-day length. This may provide a valuable tool to overcome the asynchronous flowering that has long plagued controlled hybridization in yam breeding programs (Zoundjihekpon et al., 1997).

An apparent paradox observed in our results was the increase in amylose content under SP conditions despite a reduction in tuber dry matter. This pattern likely reflects a shift in starch composition rather than a reduction in carbon allocation to tubers. While SP treatment promoted higher tuber yield, the biochemical pathways regulating starch biosynthesis may have altered the relative proportions of starch polymers. In particular, changes in the activity of enzymes involved in starch biosynthesis, such as granule-bound starch synthase responsible for amylose synthesis, may increase the amylose fraction independently of total starch accumulation (Zhang et al., 2021).

### 4.2 Hormonal applications modulate physiology but cannot bypass the photoperiodic gate

In stark contrast to the potent effect of photoperiod, none of the tested phytohormones induced flowering in non-flowering genotypes. This suggests that, under the conditions tested, exogenous hormonal applications alone were insufficient to induce flowering in non-flowering genotypes, a key difference from other species. For instance, in *D. rotundata*, combinations of cytokinins (BAP) and gibberellin antagonists have successfully induced flowering (Denadi et al., 2024), while in potato, GA is routinely used to promote flowering (Ghiyal et al., 2025). Our results show that in *D. alata*, these hormones play modulating, rather than inductive roles. The strong inhibitory effect of BAP, which blocked flowering even in flowering control plants, points to a potential antagonistic relationship between high cytokinin levels and floral initiation in yam, contrasting with its role in other species.

The most intriguing hormonal results relate to tuber quality, revealing a deep connection between stress/developmental hormones and carbohydrate metabolism. The high increase in tuber amylose content following STS application is particularly noteworthy. STS is an ethylene inhibitor. Ethylene is typically associated with ripening and senescence, processes that involve starch breakdown (Maltsev, 2021). By inhibiting ethylene perception, STS may have shifted tuber metabolism away from maturation processes and towards the synthesis of highly ordered, amylose starch. This finding partially aligns with Mondo et al (Mondo et al., 2023), who found STS enhanced floral output in *D. rotundata* and also affected tuber dry matter, suggesting a pleiotropic role for ethylene signaling in yam. Our data extends this, showing a direct link to starch quality. Nonetheless, the stronger effects observed in the second season may partly reflect the higher STS concentration used, which could have enhanced the inhibition of ethylene signaling.

Conversely, the application of jasmonates (JA/MEJA), known defense-related hormones (Koo, 2018), significantly suppressed amylose content. This suggests an antagonistic relationship between stress signaling and high-quality starch synthesis. It is plausible that activating defense pathways with JA diverts carbohydrate resources away from storage polymer synthesis and towards the production of defense compounds, as seen in other plant systems (Zhao et al., 2022; Cui et al., 2024; Zhang et al., 2021). Altogether, these results open new avenues for biotechnological or agronomic improvement for *D. alata* tuber quality.

### 4.3 Breeding implications and future directions

This study may provide yam breeders with two immediately applicable tools: 1) SP treatment for inducing flowering in recalcitrant germplasm, and 2) LP treatment for synchronizing flowering among diverse parental lines. The confirmed fertility of the induced flowers means that the vast, untapped genetic diversity locked within non-flowering accessions can be accessed for hybridization. This will open the door to breeding for key traits like disease resistance (Asiedu et al., 2003), improved environmental resilience and yield (Darkwa et al., 2020), and better quality (Dossa et al., 2025a, 2024), which may be present in non-flowering landraces.

The significant trade-offs observed, such as reduced yield under LP and altered tuber quality with hormonal application, highlight the need for targeted application. For instance, LP would be used in dedicated crossing blocks, not for general production. Hormones like STS could be explored not for flowering, but as an agronomic treatment to specifically enhance tuber starch quality for industrial applications.

Future research should focus on elucidating the molecular basis of these responses. Transcriptomic analysis of yam leaves under SP and LP treatments could identify key regulatory genes, such as *D. alata* orthologs of FT and CONSTANS (Asiedu et al., 2003). Understanding the molecular crosstalk between ethylene/jasmonate signaling and starch biosynthesis pathways in tubers could lead to novel strategies for improving yam quality. Finally, combining photoperiod and hormonal treatments (e.g., SP induction followed by a late-season STS spray) may offer a synergistic approach to simultaneously manage reproduction and optimize tuber quality.

Whether our findings are applicable to other yam species such as *D. rotundata* or across different yam-growing environments remains to be validated through further multi-location experiments. Genotype × environment interactions may influence the effectiveness of the protocol, and therefore broader validation will be necessary before large-scale adoption in breeding programs. Moreover, we did not achieve flower induction in all tested *D. alata* genotypes, indicating that additional factors beyond photoperiod and the hormones tested in this study contribute to flowering regulation. Although many other plant growth regulators exist, empirically screening all possible hormonal treatments and their combinations would require extremely large and complex experimental designs. This challenge is amplified by the short flowering window in yam (~2 months) and its long growth cycle (~9 months), making flowering‐induction trials time-consuming and labor-intensive. Finally, while this was beyond the scope of the present study, future work should explore whether non-flowering genotypes might confer yield or other agronomic advantages relative to those that flower regularly.

## Data Availability

The original contributions presented in the study are included in the article/**Supplementary Material**. Further inquiries can be directed to the corresponding authors.

## Supplementary material

Supplementary Figure 1 Weather conditions recorded during the 2022 and 2023 experimental periods. **(A–E)** 2022: **(A)** Air temperature, **(B)** Precipitation, **(C)** Wind speed, **(D)** Air pressure, and **(E)** Natural photoperiod. **(F–J)** 2023: **(F)** Air temperature, **(G)** Precipitation, **(H)** Wind speed, **(I)** Air pressure, and **(J)** Natural photoperiod.

Supplementary Figure 2 Experimental setup for the long photoperiod (LP) treatment. To extend the day length, plants were exposed to supplementary red light during the night. The top panel shows the field setup with LED projectors installed on poles. The bottom panel shows the application of red light at night. The lighting system consisted of six 50 W red LED projectors (COLIBRI IP66) for each replicate, emitting light with a peak wavelength of 630–640 nm and an intensity of 6000 lumens. Lights were automatically activated from 5:00 PM to 6:00 AM daily.

Supplementary Figure 3 Experimental setup for the short photoperiod (SP) treatment. To simulate a short-day length, yam plants were covered daily with an opaque black tarp supported by a low tunnel structure. The tarp was applied from 3:00 PM to 7:00 AM to artificially shorten the daylight exposure. The left panel shows a full view of the covered row, and the right panel provides a closer view of the support structure and the plants beneath.

Supplementary Figure 4 Morphological effect of gibberellic acid (GA) on *Dioscorea alata* flowers. Comparison of female flower inflorescences from the same genotype (‘74F’) under **(A)** GA treatment and **(B)** control (CK) treatment. Note the significant elongation and pronounced curvature of the pedicels in the GA-treated plant compared to the shorter, straighter pedicels of the control plant.

Supplementary Figure 5 Phytotoxic effects of 6-Benzylaminopurine (BAP) application on *Dioscorea alata* leaves. Foliar spray with BAP resulted in severe morphological abnormalities. Symptoms observed across multiple plants include leaf crinkling, cupping, deformation, and the development of marginal and laminal necrosis (dark, dead tissue). These symptoms indicate a strong phytotoxic response that was associated with the complete inhibition of flowering, even in fertile control genotypes.

Supplementary Figure 6 Confirmation of fertility in flowers induced by short-photoperiod (SP) treatment. The figure provides evidence for both male and female fertility in genotypes that flowered following SP induction. **(A)** Viability of pollen from the induced male genotype ‘Florido’. Micrographs show well-formed, stained pollen grains (indicated by arrows), a key indicator of male fertility. **(B)** Successful fruit set from a controlled cross between the induced female ‘74F’ and the induced male ‘Florido’. The image displays developing fruit capsules (arrows), confirming successful fertilization. **(C)** Additional evidence of successful hybridization, showing fruit set on the female genotype ‘Boutou’ after pollination by the male genotype ‘Belep’. Collectively, these results demonstrate that the SP treatment produces reproductively functional flowers, enabling controlled crosses.

Supplementary Table 1 Physicochemical properties of the soil at the experimental site. The table details the key characteristics, including soil texture (sand, silt, clay content), pH, organic matter, and major nutrient concentrations, of the topsoil (0–20 cm) at the CIRAD Roujol experimental station in Guadeloupe.
