# Supplementary figures and images for: Photoperiod modulation and hormonal application influence flowering, agronomic, morphological and tuber quality traits in greater yam (Dioscorea alata L.)
Source: Front Plant Sci. 2026 Apr 13;17:1773620. doi: 10.3389/fpls.2026.1773620 (PMC13110935; doi:10.3389/fpls.2026.1773620)

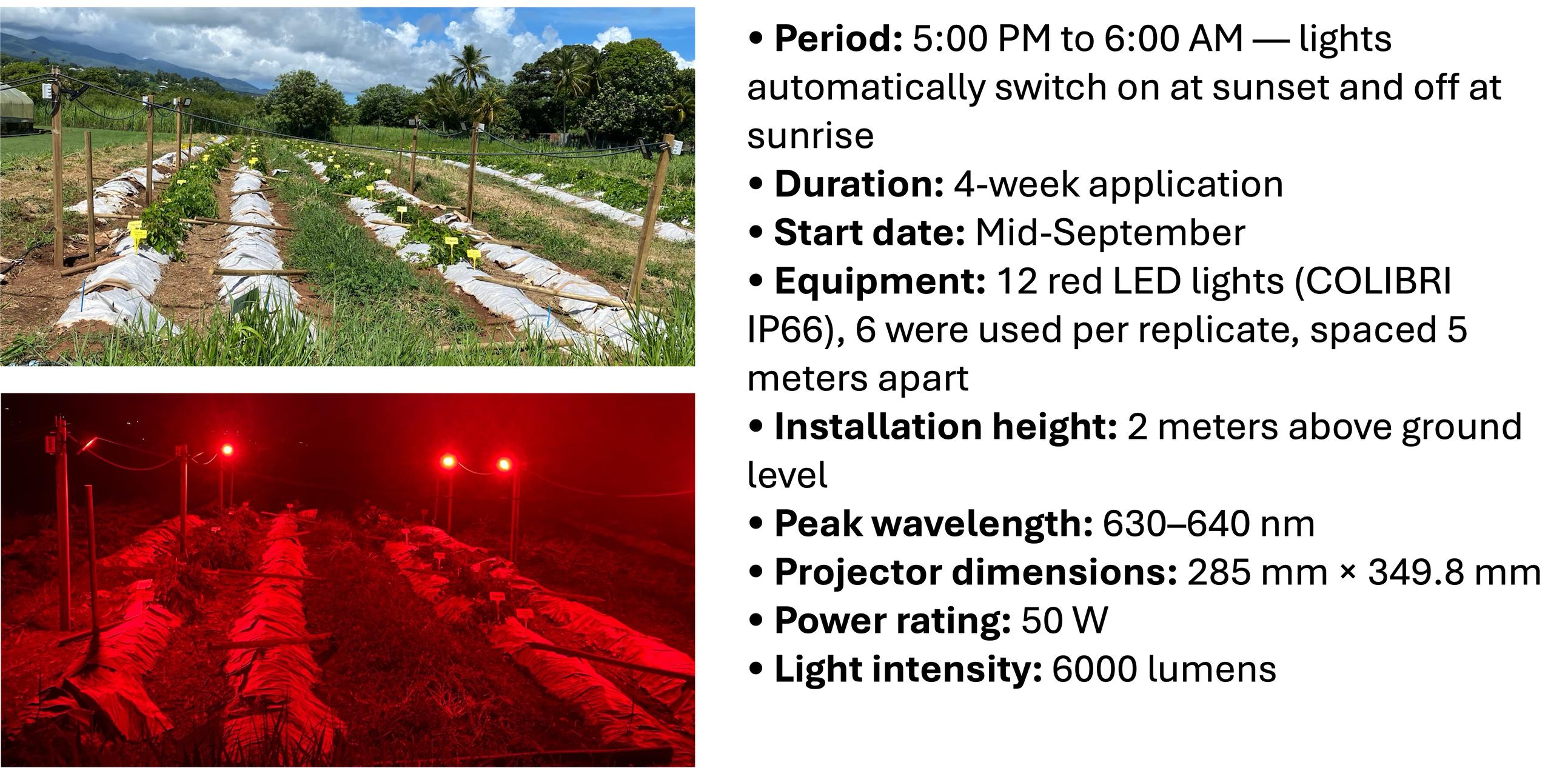

Supplement: Supplementary Figure 1 — Weather conditions recorded during the 2022 and 2023 experimental periods. (A–E) 2022: (A) Air temperature, (B) Precipitation, (C) Wind speed, (D) Air pressure, and (E) Natural photoperiod. (F–J) 2023: (F) Air temperature, (G) Precipitation, (H) Wind speed, (I) Air pressure, and (J) Natural photoperiod. [file Image1.png]

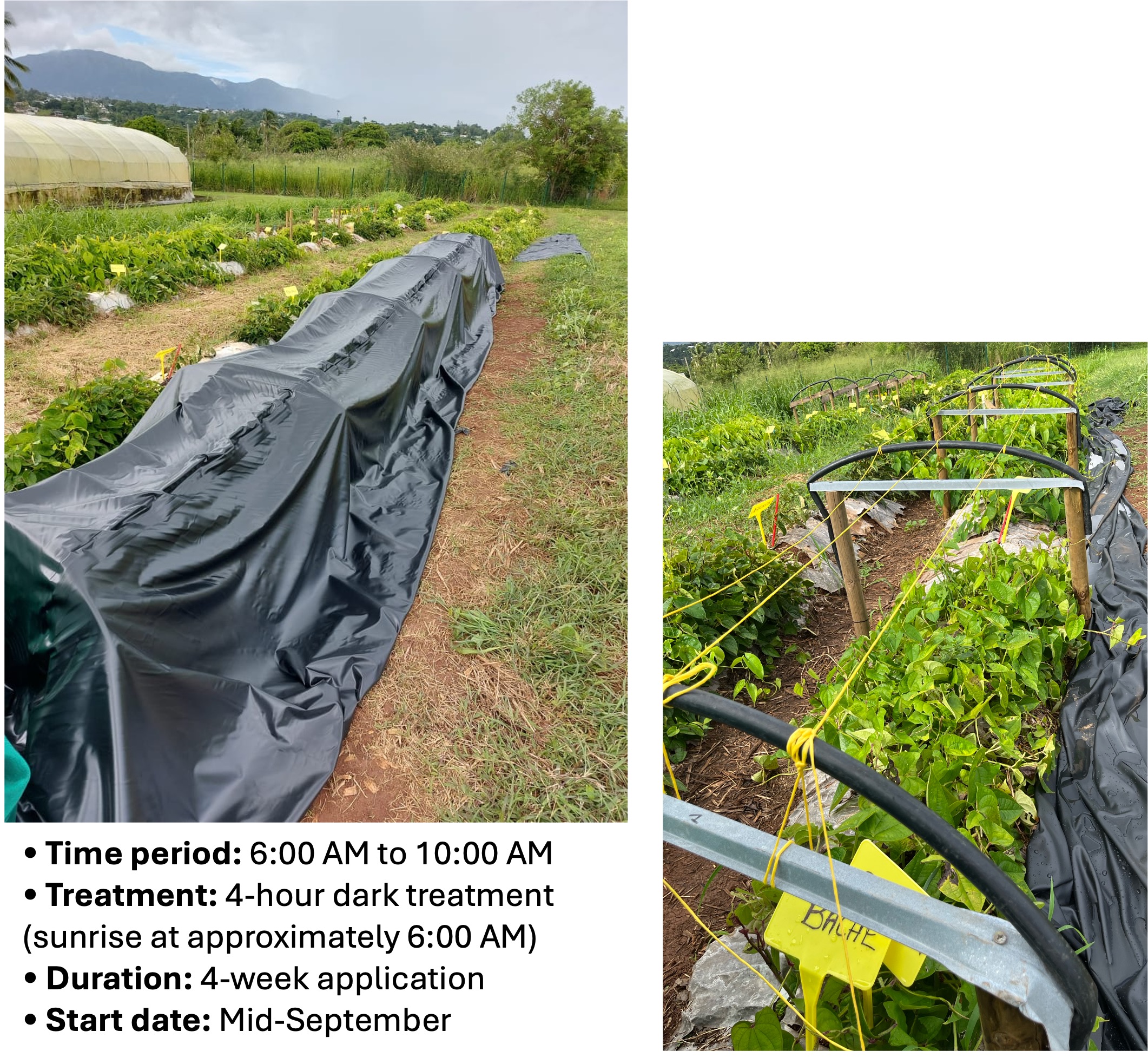

Supplement: Supplementary Figure 2 — Experimental setup for the long photoperiod (LP) treatment. To extend the day length, plants were exposed to supplementary red light during the night. The top panel shows the field setup with LED projectors installed on poles. The bottom panel shows the application of red light at night. The lighting system consisted of six 50 W red LED projectors (COLIBRI IP66) for each replicate, emitting light with a peak wavelength of 630–640 nm and an intensity of 6000 lumens. Lights were automatically activated from 5:00 PM to 6:00 AM daily. [file Image2.jpeg]

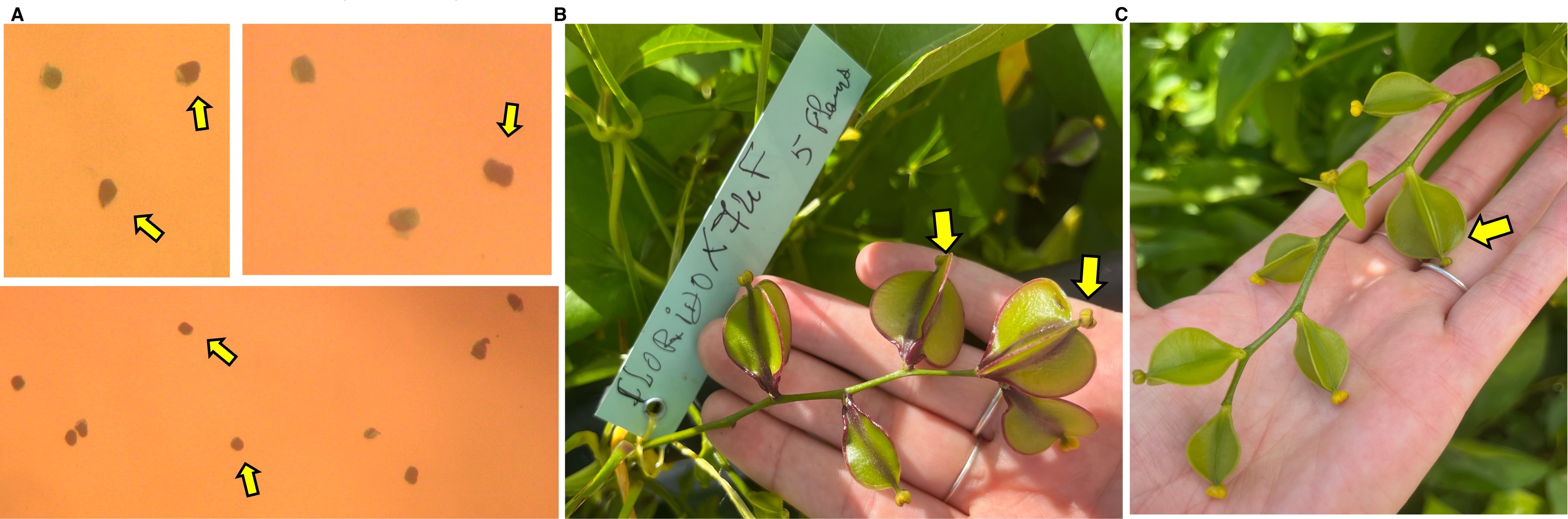

Supplement: Supplementary Figure 3 — Experimental setup for the short photoperiod (SP) treatment. To simulate a short-day length, yam plants were covered daily with an opaque black tarp supported by a low tunnel structure. The tarp was applied from 3:00 PM to 7:00 AM to artificially shorten the daylight exposure. The left panel shows a full view of the covered row, and the right panel provides a closer view of the support structure and the plants beneath. [file Image3.jpeg]

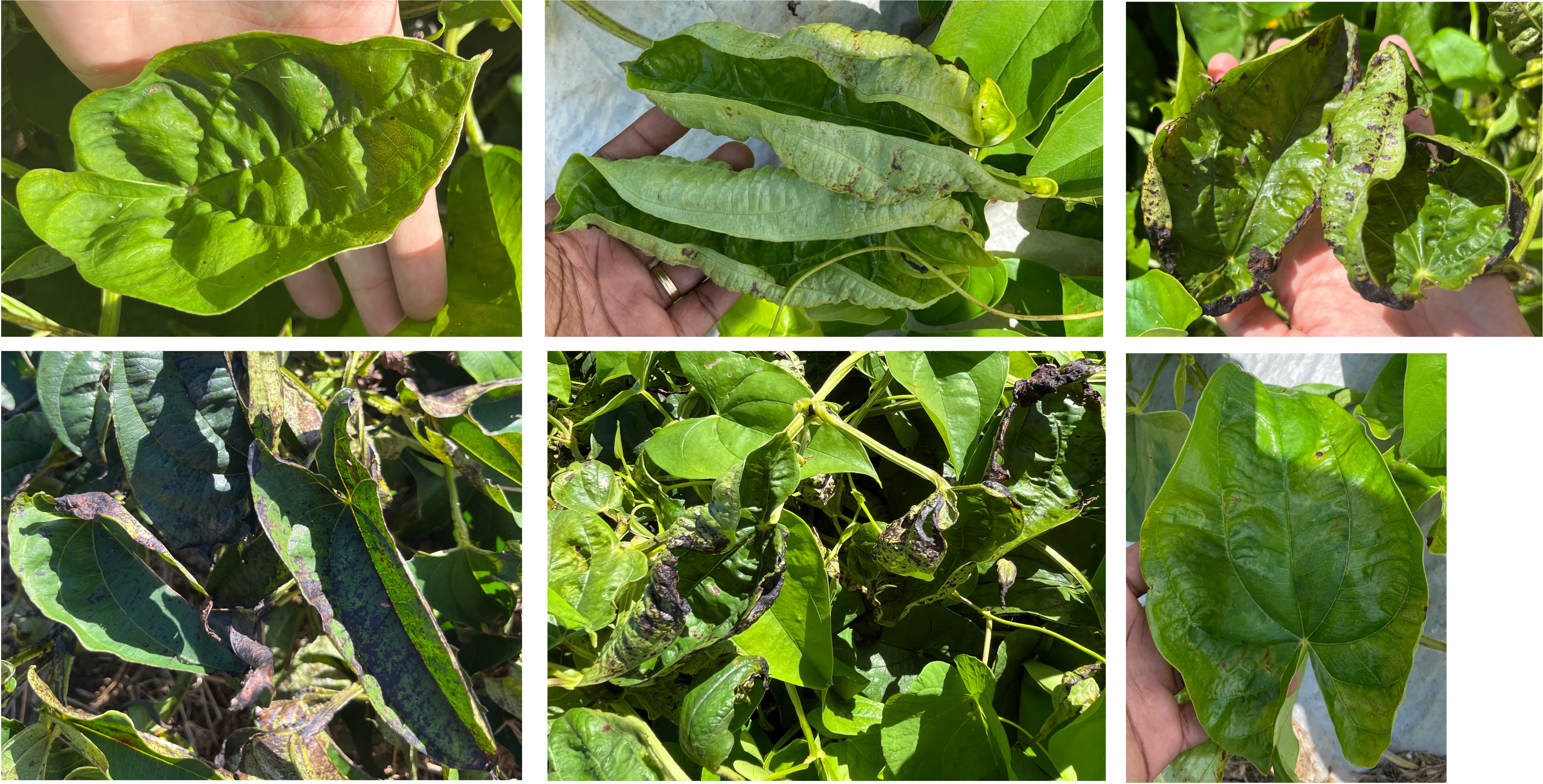

Supplement: Supplementary Figure 4 — Morphological effect of gibberellic acid (GA) on Dioscorea alata flowers. Comparison of female flower inflorescences from the same genotype (‘74F’) under (A) GA treatment and (B) control (CK) treatment. Note the significant elongation and pronounced curvature of the pedicels in the GA-treated plant compared to the shorter, straighter pedicels of the control plant. [file Image4.jpeg]

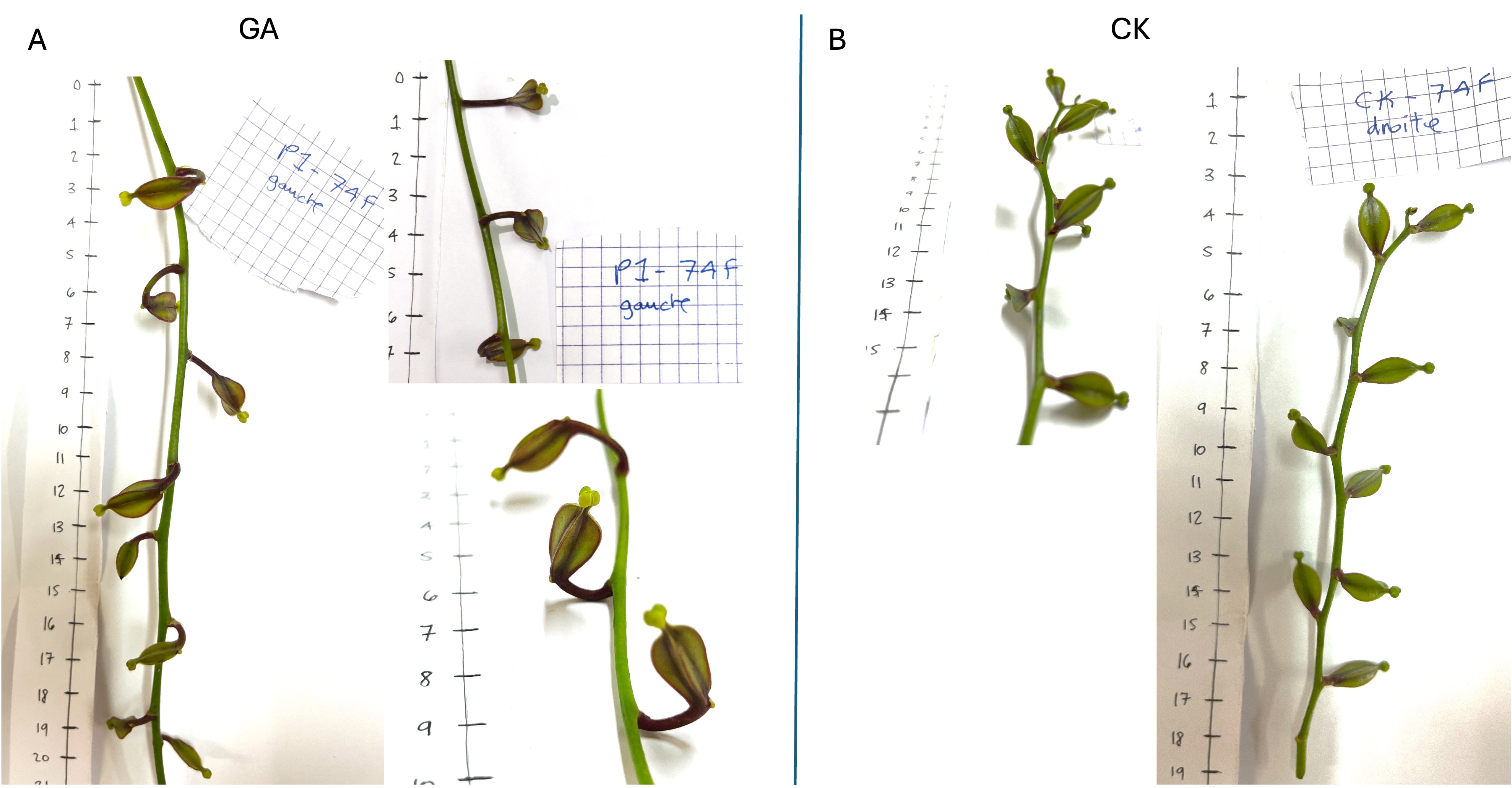

Supplement: Supplementary Figure 5 — Phytotoxic effects of 6-Benzylaminopurine (BAP) application on Dioscorea alata leaves. Foliar spray with BAP resulted in severe morphological abnormalities. Symptoms observed across multiple plants include leaf crinkling, cupping, deformation, and the development of marginal and laminal necrosis (dark, dead tissue). These symptoms indicate a strong phytotoxic response that was associated with the complete inhibition of flowering, even in fertile control genotypes. [file Image5.jpeg]
