# Supplementary material for: Photoperiod modulation and hormonal application influence flowering, agronomic, morphological and tuber quality traits in greater yam (Dioscorea alata L.)
Source: Front Plant Sci. 2026 Apr 13;17:1773620. doi: 10.3389/fpls.2026.1773620 (PMC13110935; doi:10.3389/fpls.2026.1773620)
Supplement: Supplementary Figure 6 — Confirmation of fertility in flowers induced by short-photoperiod (SP) treatment. The figure provides evidence for both male and female fertility in genotypes that flowered following SP induction. (A) Viability of pollen from the induced male genotype ‘Florido’. Micrographs show well-formed, stained pollen grains (indicated by arrows), a key indicator of male fertility. (B) Successful fruit set from a controlled cross between the induced female ‘74F’ and the induced male ‘Florido’. The image displays developing fruit capsules (arrows), confirming successful fertilization. (C) Additional evidence of successful hybridization, showing fruit set on the female genotype ‘Boutou’ after pollination by the male genotype ‘Belep’. Collectively, these results demonstrate that the SP treatment produces reproductively functional flowers, enabling controlled crosses. [file DataSheet1.pdf]

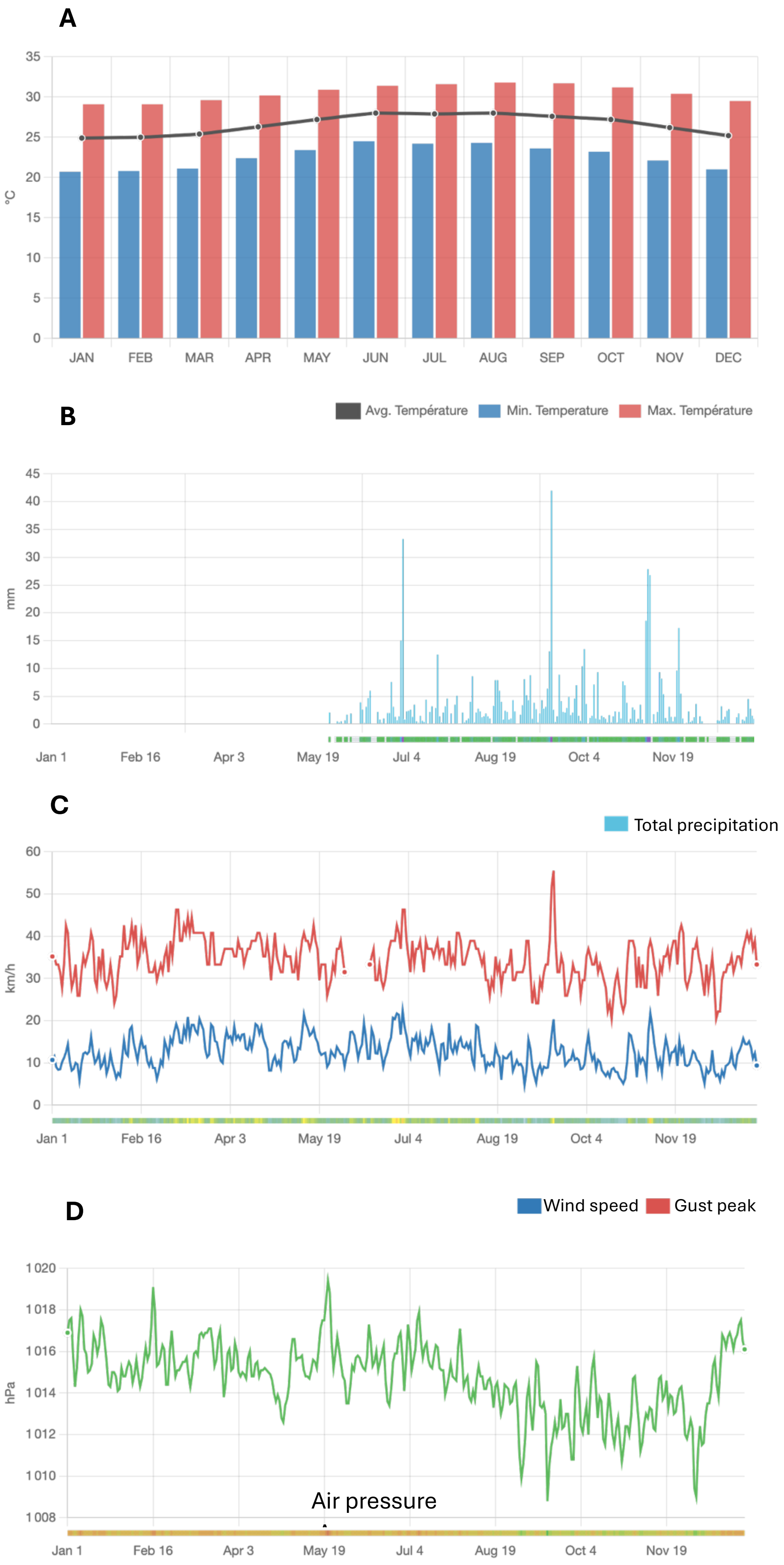
A
35
30
25
20
15
10
5
0
°C
JAN
FEB
MAR
APR
MAY
JUN
JUL
AUG
SEP
OCT
NOV
DEC
B
Avg. Température
Min. Temperature
Max. Température
45
40
35
30
25
20
15
10
5
0
mm
Jan 1
Feb 16
Apr 3
May 19
Jul 4
Aug 19
Oct 4
Nov 19
C
Total precipitation
60
50
40
30
20
10
0
km/h
Jan 1
Feb 16
Apr 3
May 19
Jul 4
Aug 19
Oct 4
Nov 19
D
Wind speed
Gust peak
1 020
1 018
1 016
1 014
1 012
1 010
1 008
hPa
Air pressure
Jan 1
Feb 16
Apr 3
May 19
Jul 4
Aug 19
Oct 4
Nov 19

E

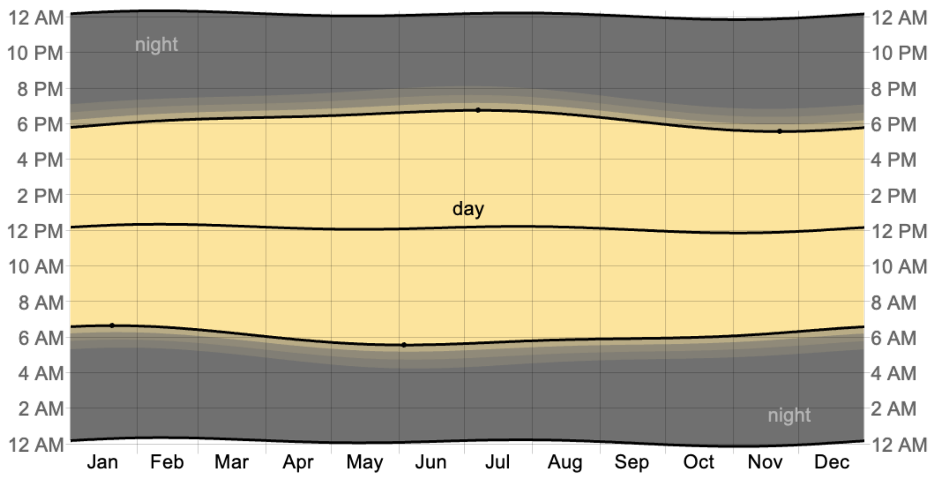
12 AM
10 PM
8 PM
6 PM
4 PM
2 PM
12 PM
10 AM
8 AM
6 AM
4 AM
2 AM
12 AM
night
day
night
Jan
Feb
Mar
Apr
May
Jun
Jul
Aug
Sep
Oct
Nov
Dec

F

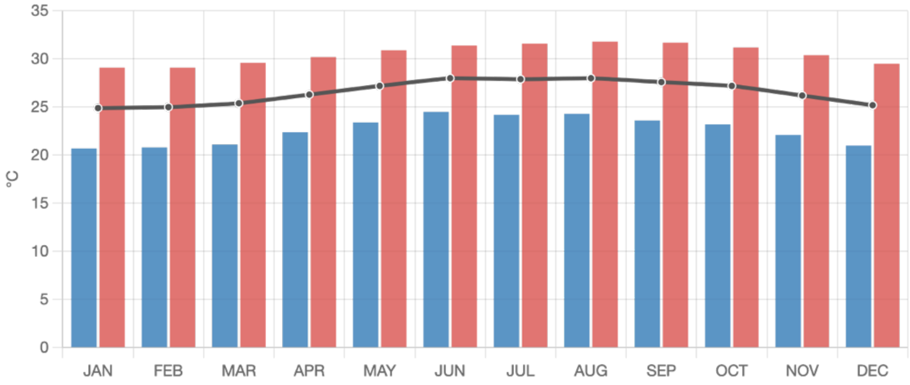
°C
35
30
25
20
15
10
5
0
JAN
FEB
MAR
APR
MAY
JUN
JUL
AUG
SEP
OCT
NOV
DEC
Avg. Température
Min. Temperature
Max. Température

G

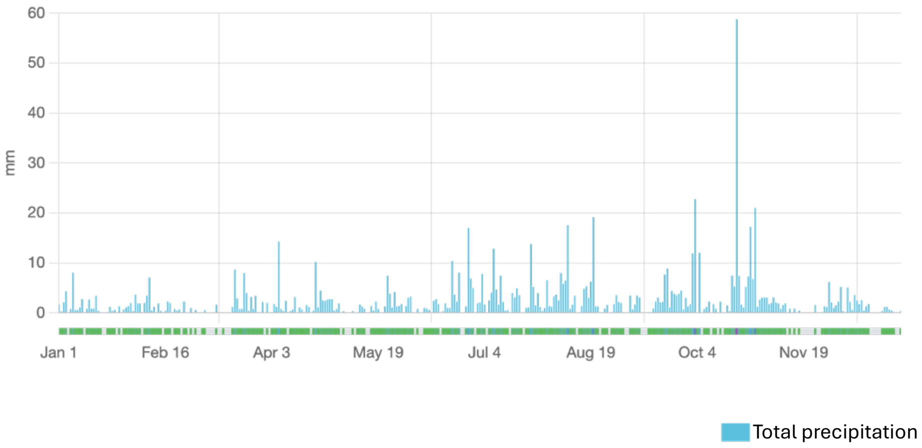
mm
60
50
40
30
20
10
0
Jan 1
Feb 16
Apr 3
May 19
Jul 4
Aug 19
Oct 4
Nov 19
Total precipitation

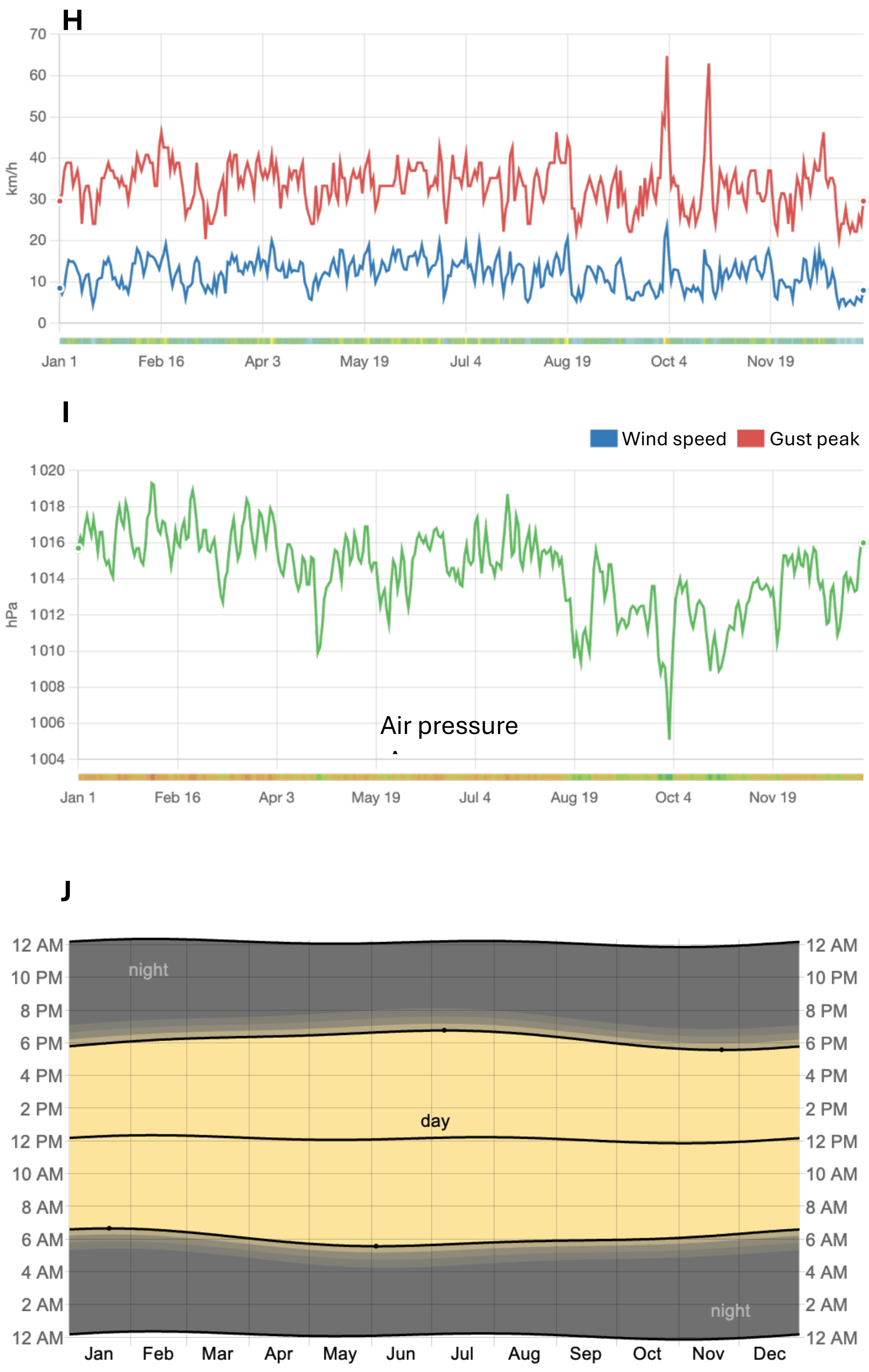

Figure S1. Weather conditions recorded during the 2022 and 2023 experimental periods.

(**A–E**) 2022: (**A**) Air temperature, (**B**) Precipitation, (**C**) Wind speed, (**D**) Air pressure, and (**E**) Natural photoperiod. (**F–J**) 2023: (**F**) Air temperature, (**G**) Precipitation, (**H**) Wind speed, (**I**) Air pressure, and (**J**) Natural photoperiod
